# Cellulose fibrils formation and organisation of cytoskeleton during encystment are essential for *Acanthamoeba* cyst wall architecture

**DOI:** 10.1038/s41598-019-41084-6

**Published:** 2019-03-14

**Authors:** Mária Garajová, Martin Mrva, Naděžda Vaškovicová, Michal Martinka, Janka Melicherová, Andrea Valigurová

**Affiliations:** 1 Department of Zoology, Faculty of Natural Sciences, Comenius University in Bratislava, 842 15 Bratislava, Slovak Republic; 2 Institute of Scientific Instruments, Czech Academy of Sciences, 612 64 Brno, Czech Republic; 3 Department of Plant Physiology, Faculty of Natural Sciences, Comenius University in Bratislava, 842 15 Bratislava, Slovak Republic; 4 Department of Botany and Zoology, Faculty of Science, Masaryk University, 611 37 Brno, Czech Republic

## Abstract

Acanthamoebae success as human pathogens is largely due to the highly resistant cysts which represent a crucial problem in treatment of *Acanthamoeba* infections. Hence, the study of cyst wall composition and encystment play an important role in finding new therapeutic strategies. For the first time, we detected high activity of cytoskeletal elements – microtubular networks and filamentous actin, in late phases of encystment. Cellulose fibrils – the main components of endocyst were demonstrated in inter-cystic space, and finally in the ectocyst, hereby proving the presence of cellulose in both layers of the cyst wall. We detected clustering of intramembranous particles (IMPs) and their density alterations in cytoplasmic membrane during encystment. We propose a hypothesis that in the phase of endocyst formation, the IMP clusters represent cellulose microfibril terminal complexes involved in cellulose synthesis that after cyst wall completion are reduced. Cyst wall impermeability, due largely to a complex polysaccharide (glycans, mainly cellulose) has been shown to be responsible for *Acanthamoeba* biocide resistance and cellulose biosynthesis pathway is suggested to be a potential target in treatment of *Acanthamoeba* infections. Disruption of this pathway would affect the synthesis of cyst wall and reduce considerably the resistance to chemotherapeutic agents.

## Introduction

Species of free-living amoebae genus *Acanthamoeba* Volkonsky, 1931 are opportunistic unicellular parasites with worldwide distribution in diverse environments including freshwater, soil, man-made habitats and even clinical settings^1–4^. Pathogenic strains are causative agents of usually fatal chronic granulomatous amoebic encephalitis (GAE) and disseminating diseases in immunodeficient individuals and *Acanthamoeba* keratitis (AK), a painful progressive eye disease in immunocompetent individuals. The constantly rising number of cases of amoebic keratitis is connected with the increasing use of contact lenses and improving awareness^5^. To date, not any standard and reliable therapeutic procedures of *Acanthamoeba* infections have been developed. The treatment of GAE and disseminated infections is limited and only rarely successful^6,7^. AK is treated with a series of drugs with various and inconsistent effects, easily manageable treatment is still not available^8,9^.

The life cycle of *Acanthamoeba* spp. comprises two stages: an active trophozoite and a dormant, metabolically almost inactive cyst. Trophozoite is a motile stage typical with hyaline spiny subpseudopodia – acanthopodia, produced on the leading pseudopodium and on the entire cell surface^10,11^. In unfavourable environmental conditions or in tissues during persistent infections trophozoites encyst^12–14^. Cyst stage is typical with a conspicuous double-layered cyst wall, consisting of ectocyst, composed mostly of proteins and polysaccharides, and endocyst, composed mostly of cellulose^15,16^. Except for cyst pores (ostioles), these layers are separated by an inter-cystic space where scattered fibrillar elements are deposited^17,18^. During the trophozoite – cyst differentiation, besides trophozoite and mature cyst, further two morphological stages were determined by Chávez-Manguía *et al*.^19^: from trophozoite developed precyst with reduced acanthopodia and immature cyst with the ectocyst.

As in *Acanthamoeba* trophozoites the permanent and distinct identification features are lacking, traditional classification of species was based exclusively on the cyst morphology, where three groups were established^20^. Group I consists of species with large cysts (diameter over 18 μm) with smooth or gently wrinkled ectocyst and stellate endocyst widely separated. Group II consists of species with cysts usually with diameter up to 18 μm with wrinkled ectocyst and stellate, polygonal, triangular, or oval endocyst, closely apposed or widely separated. This group includes the majority of described species and also the majority of species associated with human infections. Group III consists of species with diameter up to 18 μm with thin smooth or slightly wrinkled ectocyst and oval or slightly angular endocyst^11^.

The present classification of the genus *Acanthamoeba* is based on 18S rDNA and includes 21 genotypes to date (T1–T21), from which the most frequently associated with human infections is the T4 genotype^21–23^. Although both the classification approaches are not well coordinated, the morphological classification has proved useful in interpreting molecular clustering of *Acanthamoeba* isolates^24^. At present the data of thorough light microscopic and ultrastructural analyses are absenting in most of the described species and deposited strains, and prevent comprehensive comparison of morphology in particular species. To date, detailed cytomorphological data on cysts or encystment including electron microscopic methods were published only for *A. castellanii* Neff strain^17^ and clinical isolate^16,19,25^, environmental strain of *A. palestinensis*^26^ and *A. astronyxis*^27^, and for clinical isolate of *A. polyphaga*^18^. The tridimensional examination of *Acanthamoeba* cyst wall using freeze-techniques combined with transmission electron microscopy was performed only in three works focusing on *Acanthamoeba* sp^28^, *A. castellanii*^29^ and *A. polyphaga*^18^. In other species, such data are still lacking.

Highly dynamic cytoskeleton in *Acanthamoeba* is responsible for the trophozoite motility, including formation of acanthopodia, phagocytosis and first phases of the cytopathic effect in invaded tissues^30,31^. Unfortunately, the information on the role of cytoskeletal proteins tubulin, actin and a motor protein myosin is available only for trophozoites^32–36^ and the situation in cysts remains almost unknown. Inhibitor studies suggested involvement of cytoskeletal rearrangement in *Acanthamoeba* encystment^37^ and three actin-binding proteins were identified during the cyst wall formation implying actin dynamics in the course of encystment^38^. However, in mature cysts the presence of actin was not established^19^.

A further dimension is that cysts represent a serious problem in the treatment of *Acanthamoeba* infections as thanks to their wall organisation with a high content of cellulose they are highly resistant to various biocides (disinfectants, chemotherapeutic agents)^39–41^. This leads to complicated therapeutic procedures and to relapses in some cases^42^. Our understanding of the biochemistry and cell biology of the cysts is still limited and results in serious difficulties in elimination of acanthamoebae in invaded tissues^14,43^. Therefore, both the taxonomical relevance of *Acanthamoeba* cysts and their relevance as a target for treatment make them important objects for study^18,44^. In this sense particularly the study of encystment and cyst wall composition is an important topic that may play a role in finding of new strategies against *Acanthamoeba*. Cellulose is considered to be responsible for *Acanthamoeba* biocide resistance. Therefore, cellulose biosynthesis pathway is considered to be a potential target in the treatment of *Acanthamoeba* infections at present^44–46^. Results of experiments showed that the silencing of various components of this pathway, e.g. glycogen phosphorylase^47^ and cellulose synthase^48^ substantially blocked the encystment and reduced the resistance to detergents.

The aim of the present study was to characterise comprehensively the cytomorphological features and the ultrastructure of cysts and encystment in a model of the most common combination in human *Acanthamoeba* infections – T4 genotype and Group II species represented by clinical isolates of *Acanthamoeba lugdunensis* and *Acanthamoeba quina*. Various methods of the light, confocal laser scanning and electron microscopy, including SEM, TEM and freeze-etching were used providing unique detailed data and new insight into the cyst ultrastructure obtained by the tridimensional examination.

## Results

### Light microscopic and fluorescent analyses of live cysts

The cysts of T4 genotype strains of *Acanthamoeba lugdunensis* and *Acanthamoeba quina* exhibited morphology typical for Group II. On the agar plates, they were situated separately, or more frequently, in large clusters of several dozens or hundreds of individuals lying in a single plane.

**Figure 1:**
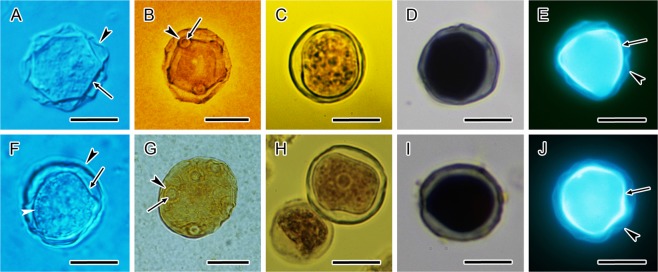
Light and fluorescence microscopy of *Acanthamoeba lugdunensis* and *Acanthamoeba quina* cysts. (**A**–**E**) *A. lugdunensis*, (**F–J**) *A. quina*. (**A**,**F**) Live cysts in differential interference contrast. The wrinkled ectocyst (arrowhead, **A**,**F**) is clearly separated from the endocyst (arrow, **A**,**F**). Cortically located lipid droplets forming a continuous layer under the cytoplasmic membrane (white arrowhead, **F**). **(B**,**G**) Cysts after silver impregnation with conspicuous circular ostioles (arrowheads, **B**,**G**) covered by opercula (arrows, **B**,**G**). (**C**,**H**) Iodine-potassium iodide stained cysts with expected no colour reaction. (**D**,**I**) Cysts after iodine-potassium iodide-sulphuric acid test with dark blue colour proving cellulose in the cyst wall. (**E**,**J**) Rylux stained mature cysts with both layers of the cyst wall. A wrinkled ectocyst (arrowheads) demonstrated faint fluorescence in comparison with an intense signal from polygonal endocyst (arrows). Bars, 10 μm.

Under light microscope the cysts of both strains were spherical or round, sometimes slightly deformed, with a wide range of size variability reaching 11.9–22.6 μm (mean 15.4 μm) in *A. lugdunensis* (Fig. 1A–E), and 12.8–18.0 μm (mean 14.9 μm) in *A. quina* (Fig. 1F–J), in diameter (Table 1). In native preparations, the outer cyst wall, the ectocyst was wrinkled and the inner cyst wall, the endocyst was polygonal with a tendency to spherical shape. The interference contrast clearly demonstrated the difference in thickness where endocyst has been showed to be thicker (Fig. 1A). The ectocyst was conspicuously separated from the endocyst, except for the region of rather indistinctive cyst pores (ostioles) where they met. In live cysts, a single spherical nucleus with a central nucleolus was situated in the central part of the cell. In the finely granular cytoplasm closely under the cytoplasmic membrane, a continuous layer of small spherical lipid droplets was noted by the interference contrast (Fig. 1F). The silver impregnation considerably enhanced the visibility of circular ostioles (5–9 per one cyst in *A. lugdunensis*, 3–5 per one cyst in *A. quina*) along with their opercula (Fig. 1B,G). With this method, the ectocyst showed no sign of a superficial reticulation in both strains. The iodine staining of cysts showed no colour reaction because cellulose does not have a helical structure and it does not bind to iodine to form a coloured product (Fig. 1C,H). The addition of sulphuric acid to the suspension of cysts mixed with iodine-potassium iodide solution proved cellulose by a very deep blue colour of both ectocyst and endocyst in both strains (Fig. 1D,I).

**Table 1:** Morphometric data on *Acanthamoeba* cysts.

	*Acanthamoeba lugdunensis*		*Acanthamoeba quina*	
	Cyst diameter^a^	Number of ostioles^b^	Cyst diameter^c^	Number of ostioles^d^
Min (μm)	11.9	5	12.8	3
Max (μm)	22.6	9	18.0	5
Mean (μm)	15.4	6.7	14.9	4.3
M (μm)	14.4	7	14.9	4
SD (μm)	3.4	0.9	1.1	0.7

Abbreviations: Min – minimum, Max – maximum, Mean – arithmetic mean, M – median, SD – standard deviation. ^a,c^Data measured on 25 cysts. ^b^Data measured on 17 cysts. ^d^Data measured on 18 cysts.

Fluorescent staining of vital mature cysts with Rylux (a diaminostilbenedisulphonic acid derivative (Mol. wt. 804 g/mol), a fluorescent optical brightener, of similar properties to Calcofluor White) displayed the clearly distinguishable ectocyst and endocyst (Fig. 1E,J). In both strains the endocyst was typical with distinct fluorescence of cellulose containing structure; however, the fluorescence in the ectocyst was faint.

### Immunofluorescent and direct fluorescent analyses of cytoskeleton and motor protein myosin

**Figure 2:**
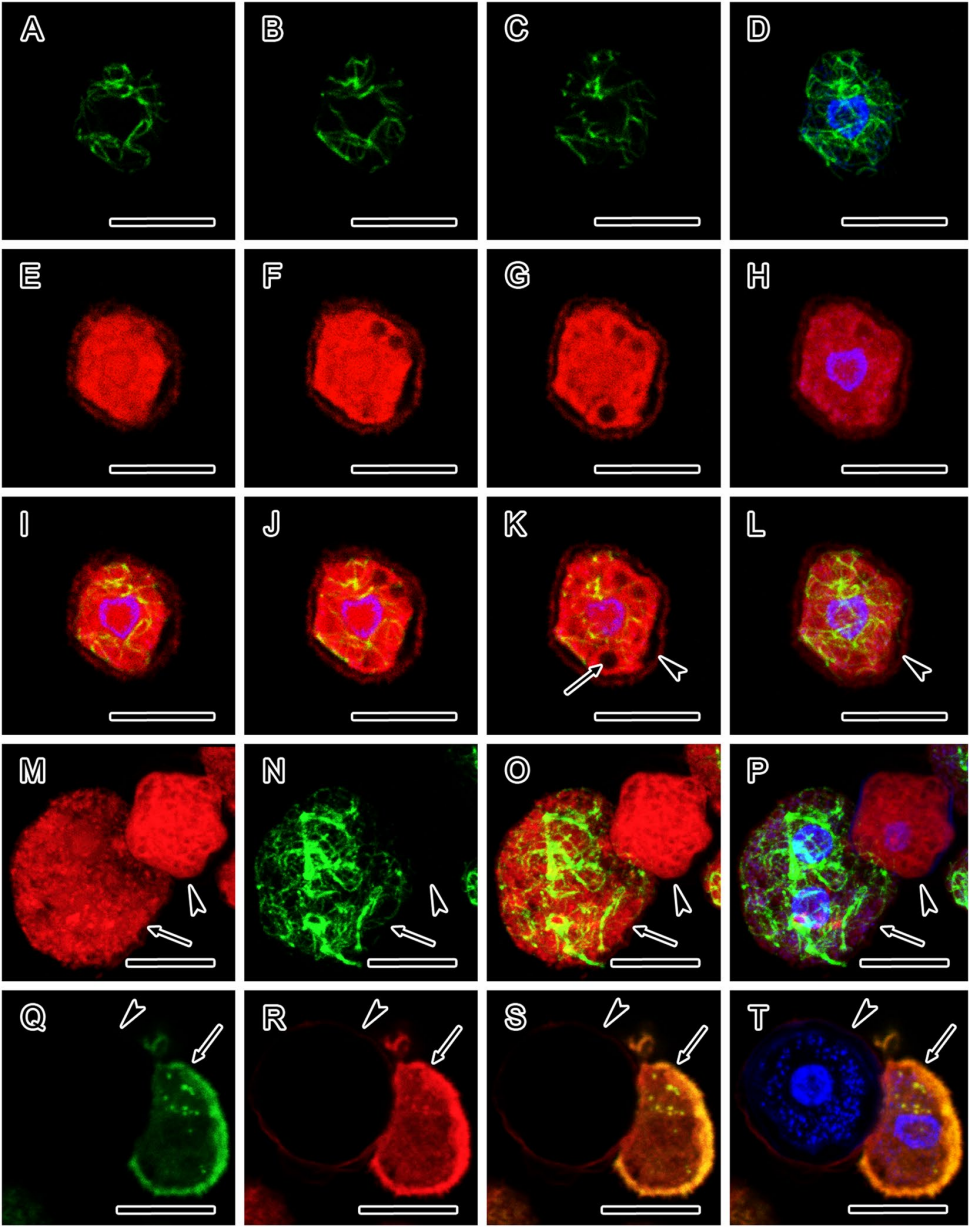
Organisation of cytoskeleton and motor protein myosin in *Acanthamoeba lugdunensis* cysts revealed by confocal laser scanning microscopy. (**A–L**) Immature cyst double-labelled with anti-α-tubulin antibody and phalloidin-TRITC showing contractile vacuole (arrow, **K**) and formed ectocyst (arrowheads, K and L). (**A–C**) Confocal sections through a cell labelled for α-tubulin, showing an irregular microtubular network. (**D**) Distribution of microtubules in immature cyst. Merged data showing FITC (anti-α-tubulin) and UV (Hoechst) fluorescence channels. (**E–G**) Confocal sections through a cell with TRITC-phalloidin labelling displaying a diffuse and patchy distribution of F-actin throughout the entire cytoplasm. (**H**) Distribution of F-actin in an immature cyst. Merged data showing TRITC (phalloidin) and UV (Hoechst) fluorescence channels. **(I–K**) Merged confocal sections through a cell showing FITC (anti-α-tubulin), TRITC (phalloidin) and UV (Hoechst) fluorescence channels. (**L**) Distribution of microtubules and F-actin in an immature cyst. Merged data showing FITC (anti-α-tubulin), TRITC (phalloidin) and UV (Hoechst) fluorescence channels. (**M–P**) Mature cyst with completed cyst wall (arrowheads, **M–P**), double-labelled with anti-α-tubulin antibody and phalloidin-TRITC. (M) Merged confocal sections through a mature cyst (arrowhead) and *Acanthamoeba* trophozoite (arrow) showing patchy presence of F-actin. TRITC (phalloidin) fluorescence channel. (**N**) Merged confocal sections through a mature cyst (arrowhead) showing absence of microtubules, which are present in *Acanthamoeba* trophozoite (arrow). FITC (anti-α-tubulin) fluorescence channel. (**O**) Merged confocal sections through a mature cyst (arrowhead) and trophozoite (arrow) showing FITC (anti-α-tubulin) and TRITC (phalloidin) fluorescence channels. (**P**) Distribution of F-actin in a mature cyst (arrowhead) with negative labelling with anti-α-tubulin and positive labelling in trophozoite cell (arrow), showing FITC (anti-α-tubulin), TRITC (phalloidin) and UV (Hoechst) fluorescence channels. (**Q–T**) Mature cyst (arrowheads) double-labelled with anti-actin and anti-myosin antibodies. (**Q**) Mature cyst (arrowhead) lacking the monomeric form of actin, which is present in a trophozoite (arrow). Merged confocal sections showing FITC (anti-actin) fluorescence channel. (**R**) Mature cyst (arrowhead) with absence of myosin, which is present in a trophozoite (arrow). Merged confocal sections showing TRITC (anti-myosin) fluorescence channel. (**S**) Merged confocal sections through a mature cyst (arrowhead) and trophozoite (arrow) showing FITC (anti-actin) and TRITC (anti-myosin) fluorescence channels. (**T**) Double-labelled cyst (arrowhead) showing the absence of both myosin and monomeric form of actin, both of which are demonstrated in *Acanthamoeba* trophozoite (arrow), showing FITC (anti-actin), TRITC (anti-myosin) and UV (Hoechst) fluorescence channels. In figures (**D**,**H**,**I**–**L**,**P**,**T**) the localisation of nuclei is displayed by Hoechst. The distance between the confocal sections in (**A**–**C**,**E**–**G**) and (**I**–**K**) is 1.0 μm. The accumulation of data in each of the figures (**D,H,L–T**) originated from 15 optical sections taken at distances of 1.0 μm. Bars, 10 μm.

**Figure 3:**
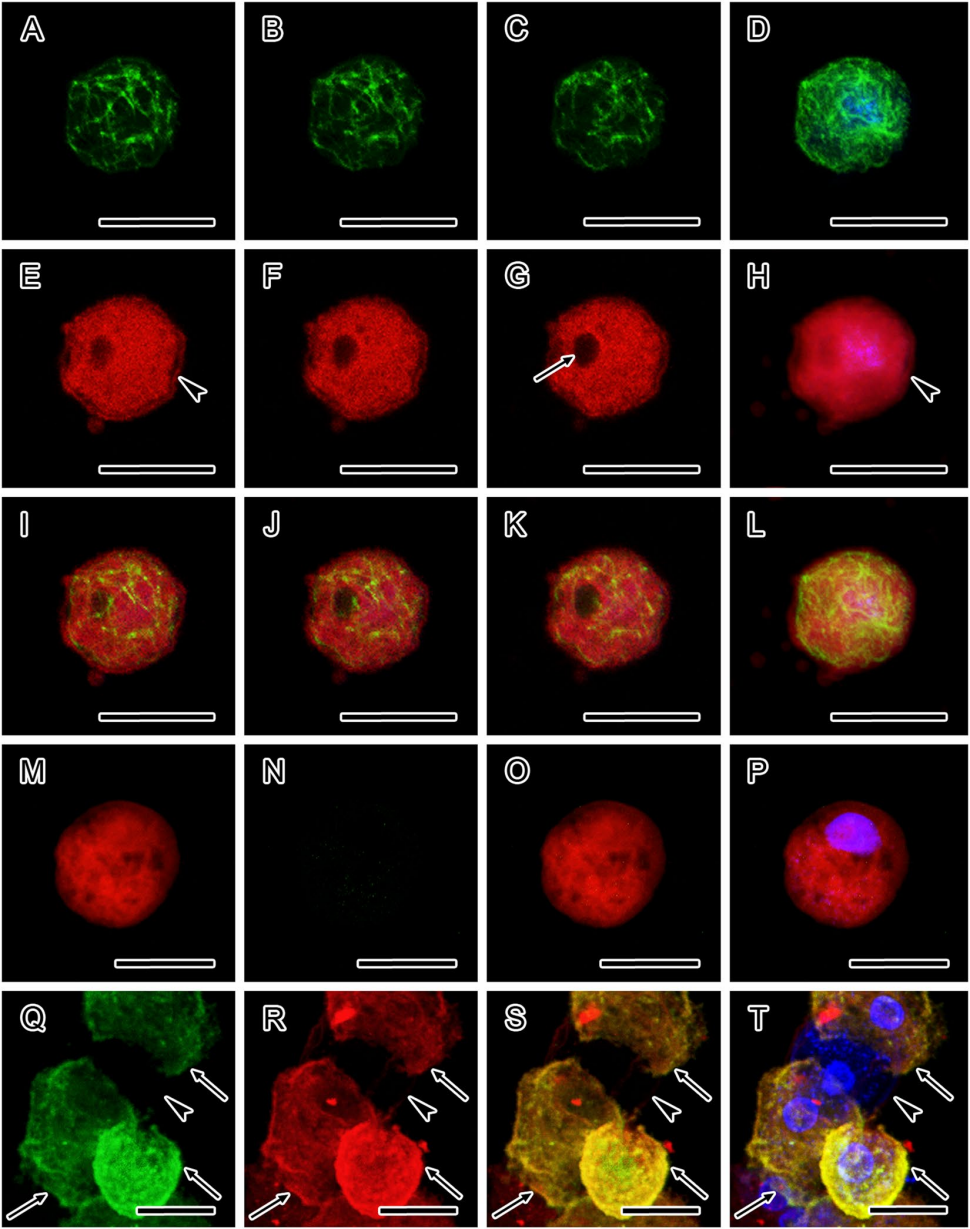
Organisation of cytoskeleton and motor protein myosin in *Acanthamoeba quina* cysts revealed by confocal laser scanning microscopy. (**A–L**) Immature cyst double-labelled with anti-α-tubulin antibody and phalloidin-TRITC showing contractile vacuole (arrow, **G**) and formed ectocyst (arrowheads, E and H). (**A–C**) Confocal sections through a cell labelled for α-tubulin, showing an irregular microtubular network. (**D**) Distribution of microtubules in immature cyst. Merged data showing FITC (anti-α-tubulin) and UV (Hoechst) fluorescence channels. (**E–G**) Confocal sections through a cell with TRITC-phalloidin labelling displaying a diffuse and patchy distribution of F-actin throughout the entire cytoplasm. (**H**) Distribution of F-actin in an immature cyst. Merged data showing TRITC (phalloidin) and UV (Hoechst) fluorescence channels. (**I–K**) Merged confocal sections through a cell showing FITC (anti-α-tubulin), TRITC (phalloidin) and UV (Hoechst) fluorescence channels. (**L**) Distribution of microtubules and F-actin in an immature cyst. Merged data showing FITC (anti-α-tubulin), TRITC (phalloidin) and UV (Hoechst) fluorescence channels. (**M–P**) Mature cyst with completed cyst wall (arrowheads, **M–P**), double-labelled with anti-α-tubulin antibody and phalloidin-TRITC. (M) Merged confocal sections through a mature cyst showing patchy presence of F-actin. TRITC (phalloidin) fluorescence channel. (**N**) Merged confocal sections through a mature cyst showing absence of microtubules. FITC (anti-α-tubulin) fluorescence channel. (**O**) Merged confocal sections through a mature cyst showing FITC (anti-α-tubulin) and TRITC (phalloidin) fluorescence channels. (**P**) Distribution of F-actin in a mature cyst with negative labelling with anti-α-tubulin, showing FITC (anti-α-tubulin), TRITC (phalloidin) and UV (Hoechst) fluorescence channels. (**Q–T**) Mature cyst (arrowheads) double-labelled with anti-actin and anti-myosin antibodies. (**Q**) Mature cyst (arrowhead) lacking the monomeric form of actin, which is present in a trophozoites (arrows). Merged confocal sections showing FITC (anti-actin) fluorescence channel. (**R**) Mature cyst (arrowhead) with absence of myosin, which is present in a trophozoites (arrows). Merged confocal sections showing TRITC (anti-myosin) fluorescence channel. (**S**) Merged confocal sections through a mature cyst (arrowhead) and trophozoites (arrows) showing FITC (anti-actin) and TRITC (anti-myosin) fluorescence channels. (**T**) Double-labelled cyst (arrowhead) showing the absence of both myosin and monomeric form of actin, both of which are demonstrated in *Acanthamoeba* trophozoites (arrows), showing FITC (anti-actin), TRITC (anti-myosin) and UV (Hoechst) fluorescence channels. In figures (**D,H,I**–**L,P,T**) the localisation of nuclei is displayed by Hoechst. The distance between the confocal sections in (**A–C,E–G)** and (**I–K**) is 0.5 μm. The accumulation of data in each of the figures (**D,H,L)** originated from 30 optical sections taken at distances of 0.5 μm. The accumulation of data in each of the figures (**M–T**) originated from 15 optical sections taken at distances of 1.0 μm. Bars, 10 μm.

For detection of cytoskeletal elements the double-labelling for confocal laser scanning microscopy using the cytoskeletal markers was performed in the strains of *A. lugdunensis* (Fig. 2) and *A. quina* (Fig. 3). For the first time, the TRITC-phalloidin staining of encysting cells in the stage of cyst wall formation (immature cysts) and early mature cysts with a completed cyst wall demonstrated the presence of filamentous actin (F-actin) and revealed its diffuse and patchy distribution throughout the entire cytoplasm excluding the cyst wall (Figs 2E–M,O,P and 3E–M,O,P). In immature cysts, a contractile vacuole (Figs 2K and 3G) and formed ectocyst (Figs 2K,L and 3E,H) were noticeable. The labelling with anti-α-tubulin antibody of immature cysts visualised a complex irregular microtubular network (Figs 2A–D,I–L and 3A–D,I–L) which was not detected in this stage until now. In cysts with completed cyst wall due to the absence of microtubules this labelling was negative (Figs 2N–P and 3N–P). In both strains the double-labelling using the specific anti-myosin and anti-actin antibodies was negative, demonstrated the absence of myosin and monomeric form of actin in cysts in comparison to trophozoites where these cytoskeletal elements were present (Figs 2Q–T and 3Q–T). The counterstaining with Hoechst displayed well the nuclei and their localisation in the cells with intense fluorescence signal in all samples (Figs 2D,H,I–L,P,T and 3D,H,I–L,P,T).

### Scanning electron microscopy

**Figure 4:**
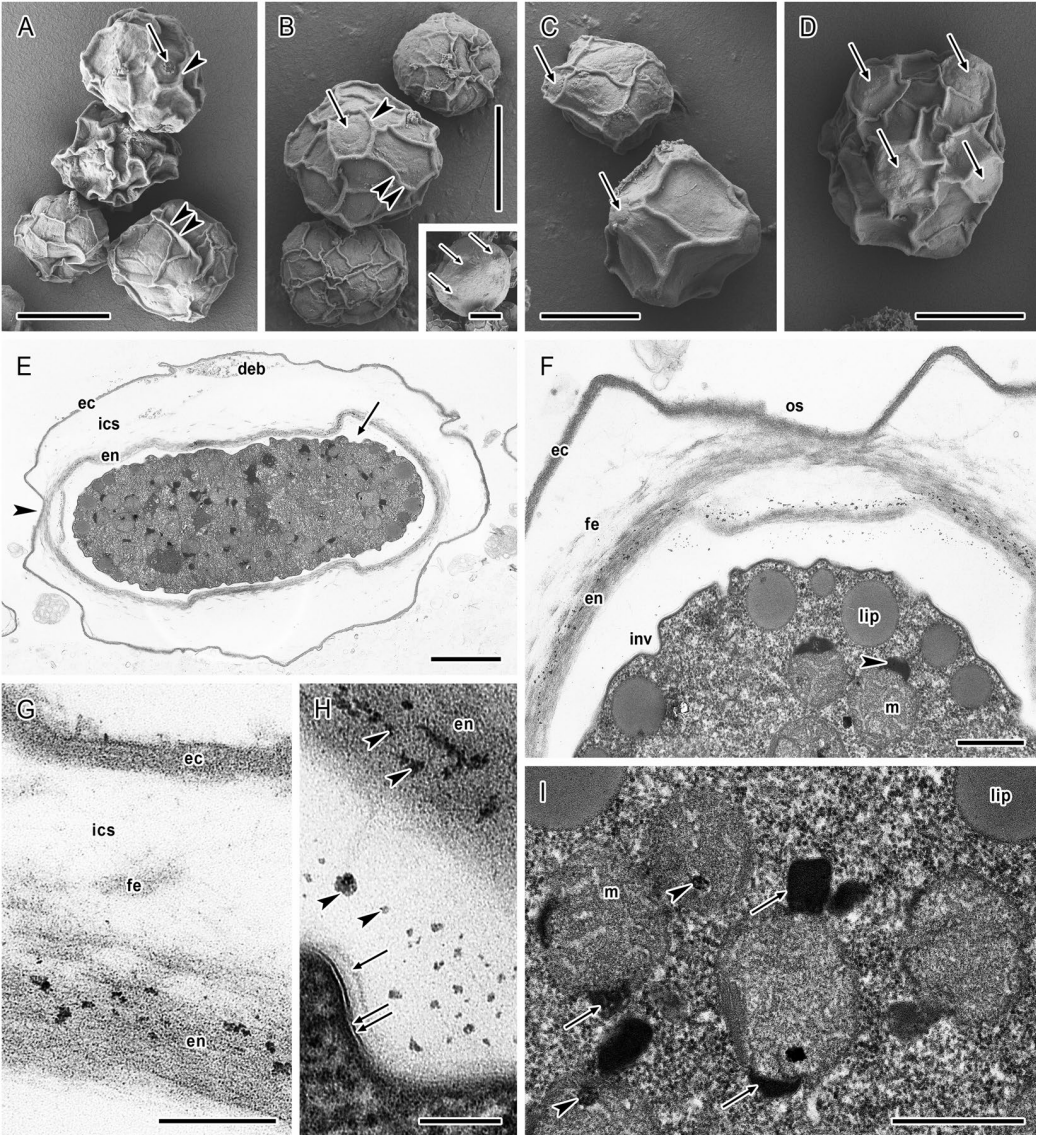
*Acanthamoeba lugdunensis* cysts architecture under scanning and transmission electron microscopy. (**A–D**) Shape and size variability in SEM. Ostioles with circular or polygonal pronounced edges (arrowheads, **A**,**B**) are covered by convex opercula (arrows, **A**–**D**). Distinct venation (double arrowheads, **A**,**B**) is typical with mostly three wrinkles joined in one point. Inset in B represents a cyst with almost unwrinkled relief with visible ostioles (arrows). (**E–I**) Cross section of cyst in TEM. (**E**) Mature cyst showing ectocyst (ec) and endocyst (en) separated by inter-cystic space (ics) with deposited cell debris (deb). Endocyst is separated from the cytoplasmic membrane of cell by an electron-transparent space (arrow). Arrowhead indicates the ostiole region. (**F**) Ostiole region of the mature cyst. The ectocyst (ec) and endocyst (en) are in the ostiole region (os) closely apposed and form a stratified continuous layer. Inter-cystic space contains deposited scattered fibrillar elements (fe). Cytoplasmic membrane of the cell forms numerous invaginations (inv). In the cytoplasm, a cortical layer of lipid droplets (lip) and spherical mitochondria (m) with peripheral electron-dense crescent-like bodies (arrowhead) are present. (**G**) Cyst wall architecture with stratified ectocyst (ec), scattered fibrillar elements (fe) deposited in inter-cystic space (ics), and loosen fibrillar structure of endocyst (en) with dispersed small electron-dense particles. (**H**) Electron-transparent space is separating endocyst (en) from the cytoplasmic membrane (double arrow) which is covered by a pale amorphous layer (arrow). Electron-dense particles (arrowheads) are deposited in both the endocyst and the electron-transparent space. (**I**) Dense cytoplasm containing lipid droplets (lip) and round mitochondria (m) with internal lamellar structures (arrowheads) and peripheral electron-dense bodies (arrows). Bars: (**A–D**, and inset) 10 μm; (**E**) 2 μm; (**F,I**) 500 nm; (**G**) 200 nm; (**H**) 100 nm.

**Figure 5:**
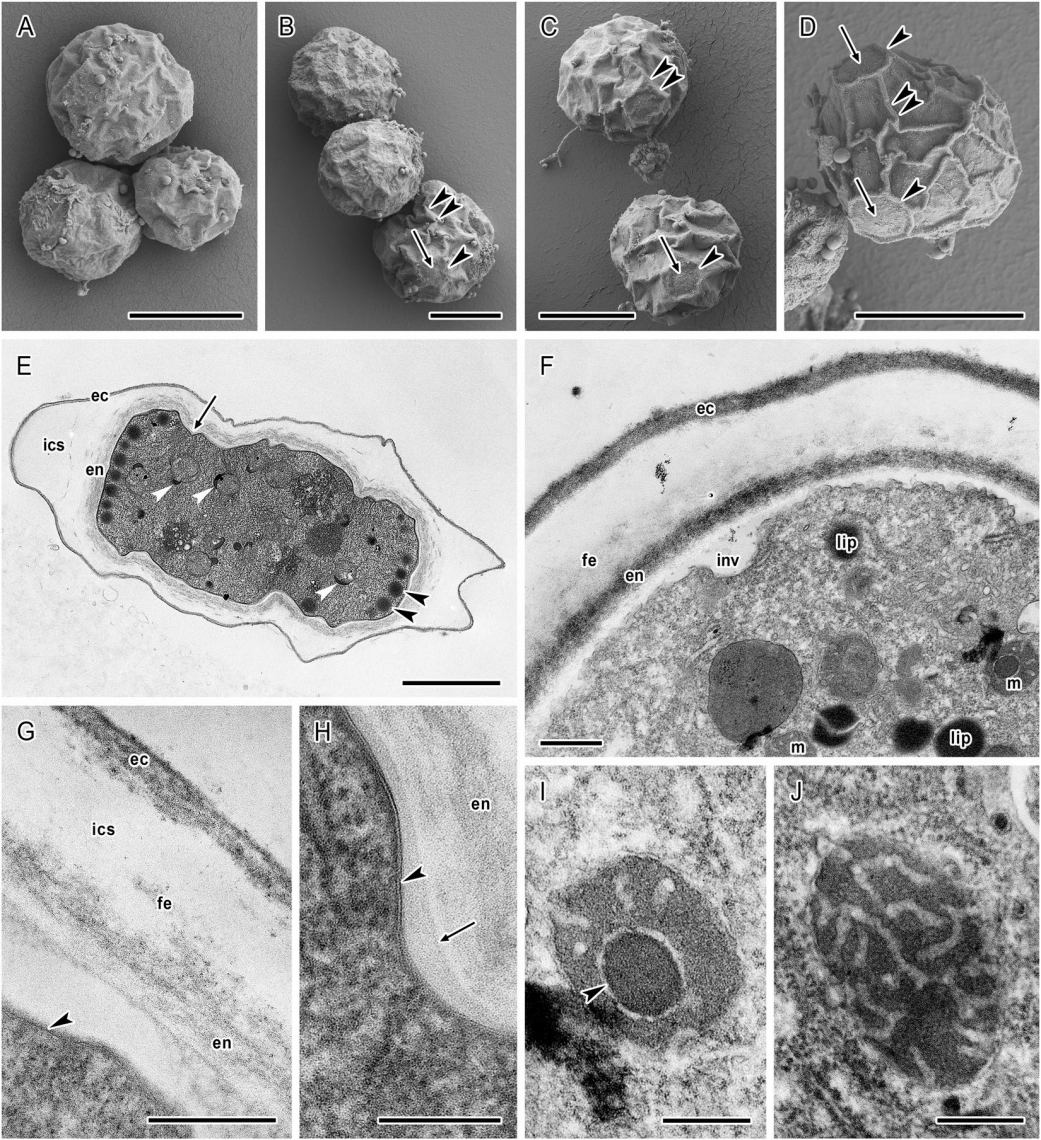
*Acanthamoeba quina* cysts architecture under scanning and transmission electron microscopy. (**A–D**) Shape and size variability in SEM. Ostioles with circular or polygonal pronounced edges (arrowheads, **B–D**) are covered by convex opercula (arrows, **B–D**). Distinct venation (double arrowheads, **B–D**) is typical with mostly three wrinkles joined in one point. (**E–J**) Cross section of cyst in TEM. (**E**) Mature cyst showing ectocyst (ec) and endocyst (en) separated by inter-cystic space (ics) with deposited scattered fibrillar elements. Endocyst is separated from the cytoplasmic membrane of cell by an electron-transparent space (arrow). Dense cytoplasm contains cortically localised lipid droplets (arrowheads) and round mitochondria with peripheral electron-dense bodies (white arrowheads). (**F**) Detail of the cross section of mature cyst. The ectocyst (ec) and endocyst (en) are separated by inter-cystic space containing deposited scattered fibrillar elements (fe). Cytoplasmic membrane of the cell forms numerous invaginations (inv). In the cytoplasm, lipid droplets (lip) and spherical mitochondria (m) are visible. (**G**) Cyst wall architecture with stratified ectocyst (ec), scattered fibrillar elements (fe) deposited in inter-cystic space (ics), and loosen fibrillar structure of endocyst (en) separated from the cytoplasmic membrane (arrowhead). (**H**) Electron-transparent space is separating endocyst (en) from the cytoplasmic membrane (arrowhead) which is covered by a pale amorphous layer (arrow). (**I,J**) Details of mitochondria in the dense cyst cytoplasm. Internal electron-dense body is indicated by arrowhead. Bars: (**A–D**) 10 μm; (**E**) 2 μm; (**F**) 500 nm; (**G–J**) 200 nm.

The surface relief in cysts of both strains under scanning electron microscopy (SEM) was typical with distinct prominent venation (Figs 4A–D and 5A–D). The thickness of wrinkles was 0.3–0.8 μm in *A. lugdunensis* strain and 0.2–0.7 μm in *A. quina* strain. In the venation, ordinarily three wrinkles joined in one point (Figs 4A–D and 5C,D). The surface wrinkles formed circular or slightly polygonal pronounced edges around the ostioles which were thus well visible. Slightly convex semispherical or crescent opercula were located in the central part of the ostioles (Figs 4A,B and 5B–D). Additionally, in *A. lugdunensis* a low number of cysts with almost unwrinkled relief but fully developed ostioles were detected in the cultures (Fig. 3B inset). Such cysts were not detected in *A. quina* strain.

### Transmission electron microscopy

In mature cysts of both strains, the ectocyst was distinctly separated from a less dense endocyst by an inter-cystic space and the endocyst was noticeably separated from the cytoplasmic membrane of the cell by an electron-transparent space (Figs 4E,F and 5E,F). Higher magnification of ultrathin sections showed a stratified structure of the ectocyst which was 65–106 nm thick in *A. lugdunensis* and 44–70 nm thick in *A. quina*, and relatively loosen fibrillar structure of the endocyst which was 210–346 nm thick in *A. lugdunensis* and 100–245 nm thick in *A. quina* (Figs 4G and 5G). In *A. lugdunensis* strain small electron-dense particles were dispersed in the endocyst and within the underneath lying space (Fig. 4H). The extensive electron-transparent inter-cystic space (2180–2400 nm thick in *A. lugdunensis* and 940–1500 nm thick in *A. quina*) contained scattered fibrillar elements deposited mainly close to the endocyst (Figs 4F,G and 5F,G). In the region of annular ostioles both the ectocyst and endocyst were closely apposed and formed a stratified continuous layer (280–300 nm thick *in A. lugdunensis*) (Fig. 4F). Ostioles were covered by opercula which were separated from the cytoplasmic membrane of the cell. In both strains the cytoplasmic membrane was 7.5–7.7 nm thick and covered by a pale, 15–55 nm thick layer. The electron-transparent space separating the endocyst from the cytoplasmic membrane was 25–539 nm thick, excluding the membrane invaginations which were 99–153 nm deep in *A. lugdunensis* and 100–403 nm deep in *A. quina* (Figs 4H and 5F). In a cortical layer of cytoplasm subjacent to the cytoplasmic membrane, lipid droplets (diameter of 202–580 nm in *A. lugdunensis* and 229–316 nm in *A. quina*) were situated (Figs 4F and 5E). Mitochondria were almost spherical to round (diameter of 456–760 nm in *A. lugdunensis* and 490–663 nm in *A. quina*), with tubular cristae (Figs 4I and 5I,J), and frequently contained eccentrically situated, electron-dense lamellar structures (55–95 nm in *A. lugdunensis* and 57–70 nm in *A. quina*). Strongly electron-dense crescent-like bodies (diameter of 131–242 nm in *A. lugdunensis* and 189–344 nm in *A. quina*) were frequently located at the periphery of mitochondria and occasionally deformed the shape of mitochondria (Figs 4I and 5E).

### Freeze-etching electron microscopy

**Figure 6:**
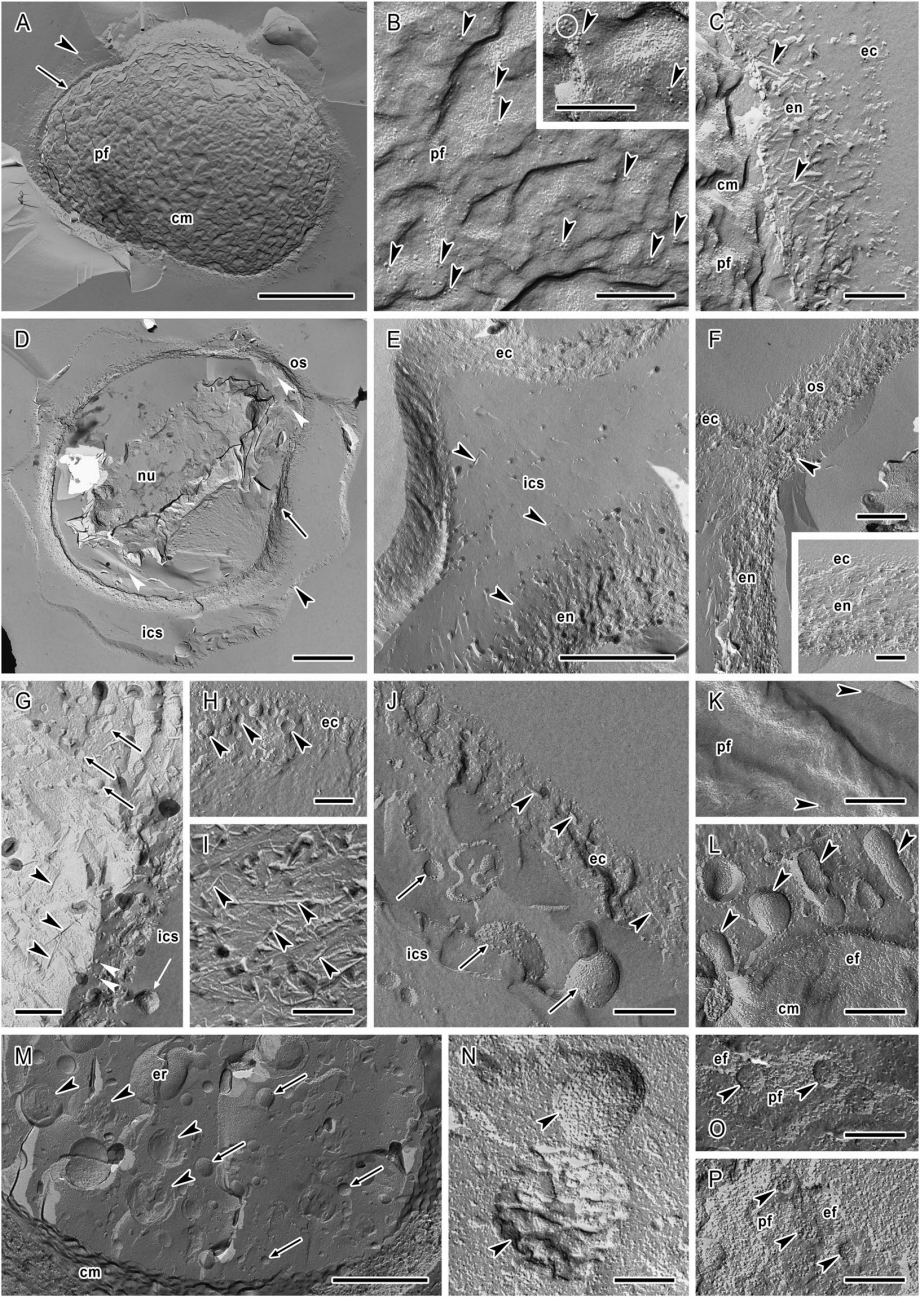
Cellular structure of *Acanthamoeba lugdunensis* cysts revealed by the freeze etching. (**A**) General view on the immature cyst showing already formed ectocyst (arrowhead) and endocyst (arrow) which is synthesised on the surface of highly wrinkled cytoplasmic membrane (cm); pf – PF of the cytoplasmic membrane. (**B**) The cytoplasmic membrane of the cell synthesising the endocyst demonstrating numerous intramembranous particles with tendency to cluster into diads and triads (arrowheads); pf – PF of the cytoplasmic membrane. The inset shows detail of the cytoplasmic membrane with one tetrad (encircled) and two triads (arrowheads) of intramembranous particles. (**C**) Cell wall in the process of the endocyst (en) synthesis on the surface of closely adherent cytoplasmic membrane (cm). Crossed cellulose fibrils in the endocyst (arrowheads), the ectocyst (ec), pf – PF of the cytoplasmic membrane. (**D**) General view on the mature cyst showing the ectocyst (arrowhead) and endocyst (arrow) separated by inter-cystic space (ics). Both layers of the cyst wall are closely apposed in the ostiole region (os). The space separating the endocyst from the cytoplasmic membrane is demonstrated as different fracture levels (white arrowheads). In the cytoplasm, the nucleus (nu) is visible. (**E**) Double-layered cyst wall composed of well differentiated ectocyst (ec) separated from the endocyst (en) by inter-cystic space (ics) containing scattered cellulose fibrils (arrowheads). (**F**) Architecture of the ostiole region (os) where thin ectocyst (ec) layer is closely associated with the underlying endocyst (en). In this region the endocyst is typical with stratified arrangement of the cellulose fibrils (arrowhead) in parts closer to the cytoplasmic membrane. The inset shows detail of the ostiole region with connection of both layers of the cyst wall. (**G**) Structure of the endocyst material with presence of cellulose fibrils (arrowheads), protein particles (white arrowheads) and spherical and oval vesicles (arrows). Vesicle situated in the inter-cystic space (ics) is indicated by white arrow. (**H**) Heterogenous structure of the ectocyst (ec) with the presence of round vesicles (arrowheads). (**I**) Endocyst structure showing unarranged crossed cellulose fibrils (arrowheads). (**J**) Ectocyst (ec) demonstrating scattered cellulose fibrils (arrowheads). Vesicles in the inter-cystic space (ics) are indicated by arrows. (**K**) Cytoplasmic membrane of the mature cyst with finished cell wall synthesis showing low number of intramembranous particles (arrowheads); pf – PF of the cytoplasmic membrane. (**L**) Cytoplasmic membrane (cm) of cyst with endocyst almost complete showing release of vesicles (arrowheads); ef – EF of the cytoplasmic membrane. (**M**) Fracture of the mature cyst cytoplasm outlined by wrinkled cytoplasmic membrane (cm). Endoplasmic reticulum (er), numerous spherical lipid droplets (arrowheads) and vesicles (arrows) are visible in entire volume of cytoplasm. (**N**) Detail of the spherical mitochondria (arrowheads) in the dense cyst cytoplasm. (**O**) Well preserved nuclear pore complexes (arrowheads) of the immature cyst; pf – PF of the inner membrane of nuclear envelope, ef – EF of the outer membrane of nuclear envelope. (**P**) Nuclear pore complexes (arrowheads) of mature cyst showing smaller size and weaker differentiation; pf – PF of the outer membrane of nuclear envelope, ef – EF of the inner membrane of nuclear envelope. Bars: (**A,D**) 2 μm; (**B**,**C,G**–**L,N**–**P**, insets in **B** and **F**) 200 nm; (**E,M**) 1 μm; (**F**) 500 nm.

**Figure 7:**
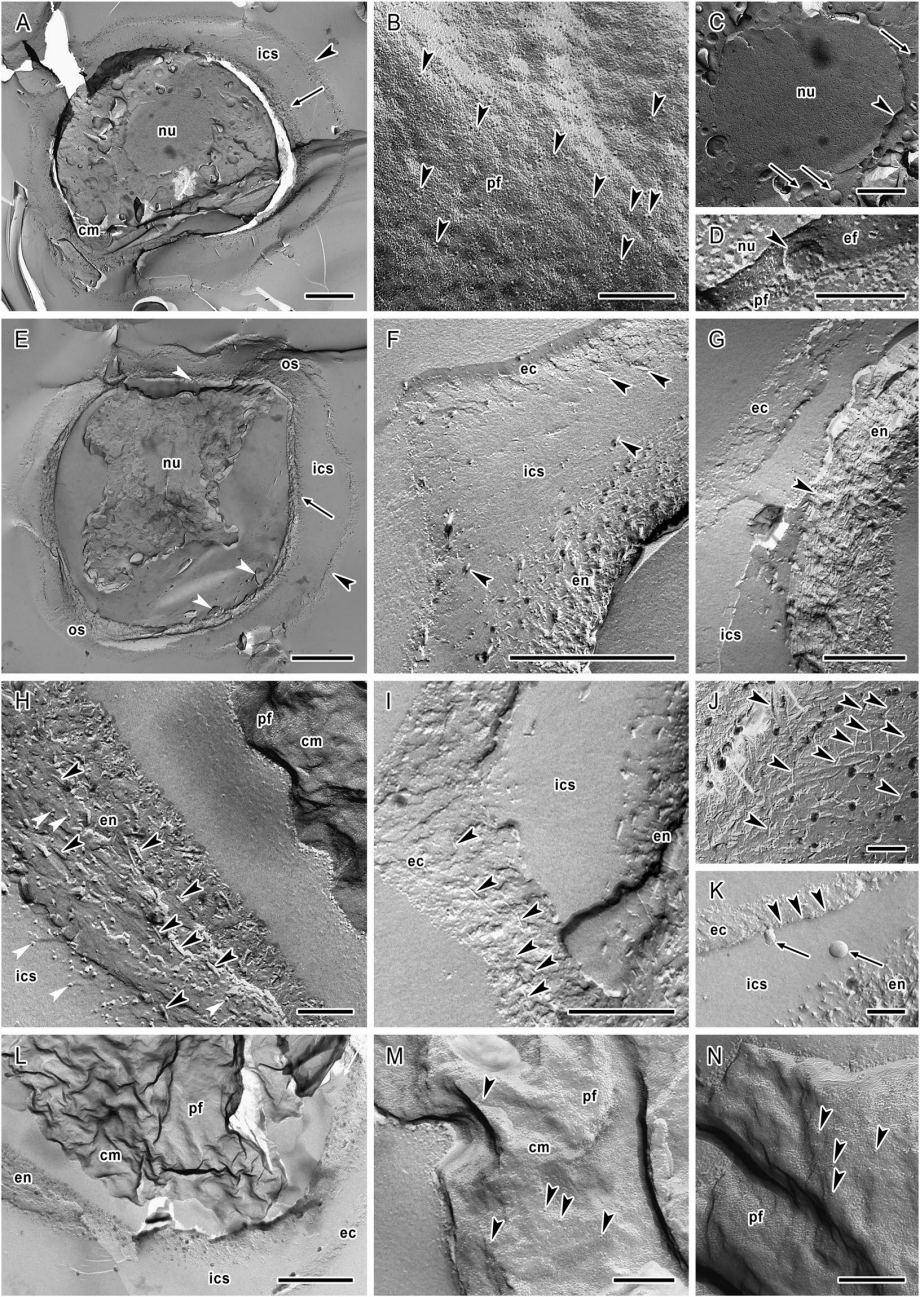
Cellular structure of *Acanthamoeba quina* cysts revealed by the freeze etching. (**A**) General view on the immature cyst showing already formed ectocyst (arrowhead) and endocyst (arrow) which is synthesised on the surface of highly wrinkled cytoplasmic membrane (cm). The ectocyst and endocyst are separated by inter-cystic space (ics). In the cytoplasm, the nucleus (nu) is visible. (**B**) The cytoplasmic membrane of the cell synthesising the endocyst showing numerous intramembranous particles with tendency to cluster into diads and triads (arrowheads); pf – PF of the cytoplasmic membrane. (**C**) Nucleus (nu) of the immature cyst with surrounding vesicles in the cytoplasm (arrows). The nuclear envelope is indicated by arrowhead. (**D**) Nuclear pore complex (arrowhead) of the nucleus (nu) of immature cyst in exoplasmic fracture face of the inner membrane of nuclear envelope (ef); pf – PF of the outer membrane of nuclear envelope. (**E**) General view on the mature cyst showing the ectocyst (arrowhead) and endocyst (arrow) separated by inter-cystic space (ics). Both layers of the cyst wall are closely apposed in the ostiole region (os). The space separating the endocyst from the cytoplasmic membrane is demonstrated as different fracture levels (white arrowheads). In the cytoplasm, the nucleus (nu) is visible. (**F**) Double-layered cyst wall composed of well differentiated ectocyst (ec) separated from the endocyst (en) by inter-cystic space (ics) containing scattered cellulose fibrils (arrowheads). (**G**) Architecture of the ostiole region where ectocyst (ec) layer is close to the underlying endocyst (en) and the inter-cystic space (ics) is reduced. The endocyst is typical with stratified arrangement of the cellulose fibrils (arrowhead). (**H**) Structure of the endocyst (en) material with presence of numerous cellulose fibrils (arrowheads) and protein particles (white arrowheads) present also in the inter-cystic space (ics). The cytoplasmic membrane (cm) is separated from the endocyst; pf – PF of the cytoplasmic membrane. (**I**) Ectocyst (ec) demonstrating scattered cellulose fibrils (arrowheads), the inter-cystic space (ics), the endocyst (en). **(J**) Endocyst structure showing unarranged crossed cellulose fibrils (arrowheads). (**K**) Ectocyst (ec) showing scattered cellulose fibrils (arrowheads). Vesicles in the inter-cystic space (ics) are indicated by arrows, the endocyst (en). (**L**) View on the highly wrinkled cytoplasmic membrane (cm) of the mature cyst; the endocyst (en), the inter-cystic space (ics), the ectocyst (ec), pf – PF of the cytoplasmic membrane. (M and N) Details of cytoplasmic membranes of mature cysts with finished cell wall synthesis showing low number of intramembranous particles (arrowheads); pf – PF of the cytoplasmic membrane. Bars: (**A,E**) 2 μm; (**B,D,H,J,K,M,N**) 200 nm; (**C,F,L**) 1 μm; (G and I) 500 nm.

Processing by the freeze-etching technique enabled an excellent preservation of internal cellular structures and enabled their tridimensional examination in cysts of strains of *A. lugdunensis* (Fig. 6) and *A. quina* (Fig. 7). Generally, the architecture of cysts corresponded to transmission electron microscopy (TEM) observations.

The ectocyst and endocyst were well preserved and their thickness corresponded with the data measured in TEM sections. The ectocyst structure was heterogenous (Figs 6E,H,J and 7F,G,I,K), however, with a well-defined outline and it possessed scattered fibrils of a spiny appearance resembling the main structural material of the endocyst in both strains (Figs 6J and 7I). These cellulose fibrils were demonstrated for the first time in *Acanthamoeba* ectocyst. In some specimens, oval or spherical vesicles of 25–86 nm were present (Fig. 6H).

The endocyst in both strains was characteristic with an extensive predominance of cellulose material in the form of crossed fibrils of spiny appearance and thickness of 7.8–17.8 nm (Figs 6C,E–G,I and 7G,H,J). The fibrils were unarranged and without any orientation, except for the ostioles region, where the cellulose material in areas closer to the cytoplasmic membrane was distributed more in layer-like manner outlining the cell shape (Figs 6F and 7G). Generally, the endocyst presented a compact character near the cytoplasmic membrane and was of more loosened appearance near the inter-cystic space (Figs 6E and 7F,I). In the endocyst material, numerous small spherical or oval vesicles (size 30–80 nm) were randomly distributed (Fig. 6G), and many tiny presumably protein particles were detected (Figs 6G and 7H).

In both strains the inter-cystic space (Figs 6D,E,J and 7A,E–G,I) contained freely scattered fibrils that seemed to originate from the loosened part of endocyst. In this space, vesicles similar to that observed in ectocyst were found occasionally (Figs 6G,J and 7K). The ostiole region showed the same structure as observed in ultrathin sections, i.e. closely apposed thin ectocyst layer and underlying endocyst (Figs 6F and 7E).

Correspondingly to the TEM, The cytoplasmic membrane was considerably wrinkled in both strains (Figs 6A–C,M and 7A,L–N) and in mature cysts mainly without releasing of vesicles from the cortical cytoplasm (Figs 6K,M and 7L–N). However, in some cytoplasm regions, the presence and release of vesicles (size 80–350 nm) was detected indicating the incompletion of encystment (Fig. 6L). In immature cysts, the cytoplasmic membrane was adherent to the endocyst in process of its formation, without any separating space (Figs 6A–C and 7A). In mature cysts, this thin space was formed and freeze-etching visualised it as different fracture levels located between the cytoplasmic membrane and endocyst (Figs 6D and 7E).

The cytoplasm of immature cysts appeared less condensed in comparison with the cytoplasm of mature cysts where numerous vesicles, lipid droplets (Fig. 6M) and spherical mitochondria (Fig. 6N) were clearly visible. Generally, in the nuclear membrane, nuclear pore complexes were well preserved. In immature cysts their size reached 77.2–83.8 nm (mean 80.7 nm) in *A. lugdunensis* strain (Fig. 6O), and 76.3–97.2 nm (mean 87.4 nm) in *A. quina* strain (Fig. 7D). In mature cysts with dehydrated nucleoplasm and nuclear membrane weakly differentiated from the cytoplasm, the nuclear pore complexes were 63.4–77.6 nm (mean 71.5 nm) in *A. lugdunensis* strain (Fig. 6P) in diameter.

**Figure 8:**
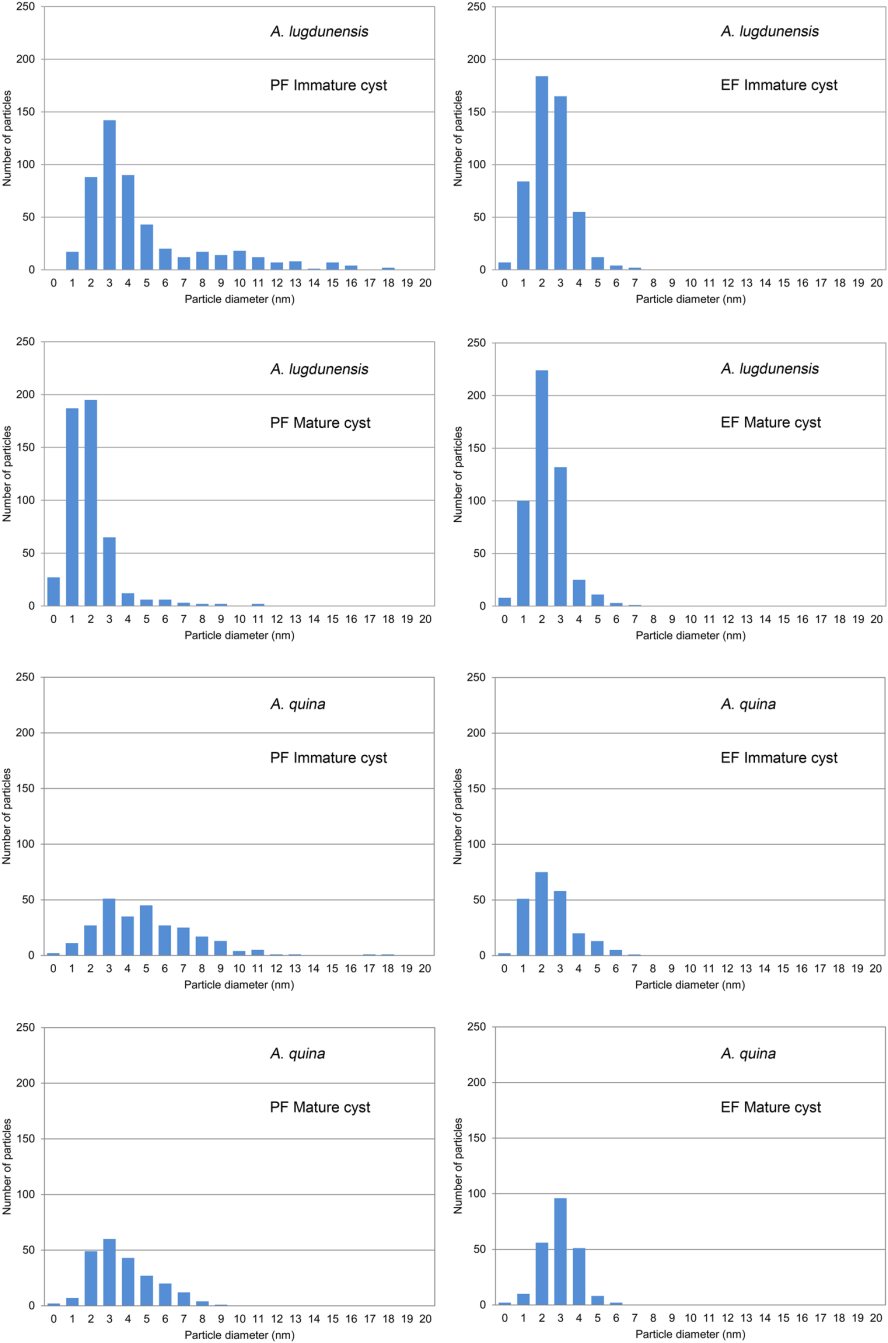
Histograms of the IMP size distribution in fracture faces of the cytoplasmic membranes of immature and mature *Acanthamoeba* cysts. Protoplasmic fracture face (PF); exoplasmic fracture face (EF).

In the cytoplasmic membrane of immature cysts of both strains, clustering of intramembranous particles (IMPs) into diads, triads, tetrads or even more was observed (Figs 6B and 7B) which was not discernible in mature cysts (Figs 6K and 7M,N). Generally, distinctly higher density of IMPs was noted in cytoplasmic membrane of immature cysts (Table 2). The number of IMPs was noticeably higher in the exoplasmic fracture face (EF) than in the protoplasmic fracture face (PF) in both the immature and the mature cysts. In *A. lugdunensis* the size of IMPs in PF decreased considerably during the encystment process from the mean 5.5 nm (SE = 0.1 nm) in immature cysts during the endocyst synthesis, to 2.4 nm (SE = 0.1 nm) in the mature cysts. Similarly, in *A. quina* the size of IMPs in PF decreased from the mean 5.5 nm (SE = 0.2 nm) in immature cysts, to 4.2 nm (SE = 0.1 nm) in the mature cysts. Simultaneously, dehydration of the cells and deformation of their cytoplasmic membranes to wrinkles led to a higher definition of IMPs and in mature cysts their density decreased to about 40% in PF and to about 25% in EF in *A. lugdunensis*, and to about 3–10% in PF and 28% in EF in *A. quina*. The information on IMPs size and density in fracture faces of cytoplasmic membranes of the immature and the mature cysts is summarised in the Table 2. Observed size variability of IMPs was high in both strains, ranging from 0.4 to 18.7 nm (see histograms in Fig. 8).

**Table 2:** Size and density of intramembranous particles (IMPs) in fracture faces of cytoplasmic membrane of *Acanthamoeba* cysts. All data represent mean values ± standard error.

	Face	*Acanthamoeba lugdunensis*		*Acanthamoeba quina*	
		Size of particles ± SE (nm)	Number of particles/μm^2^ ± SE	Size of particles ± SE (nm)	Number of particles/μm^2^ ± SE
Immature cyst^a^	PF	5.5 ± 0.1	1059 ± 77	5.5 ± 0.2	2056 ± 659
	EF	2.9 ± 0.1	7793 ± 890	2.9 ± 0.1	3074 ± 810
Mature cyst	PF	2.4 ± 0.1	443 ± 44	4.2 ± 0.1	63, 201^b^
	EF	3.0 ± 0.1	1989 ± 552	3.4 ± 0.1	872 ± 242

Abbreviations: PF – protoplasmic fracture face, EF – exoplasmic fracture face, SE – standard error. ^a^Cyst in the stage of endocyst synthesis. ^b^Data obtained from two different specimens.

## Discussion

In this study for a first time, a combined approach of the light, confocal laser scanning and electron microscopy, and freeze-etching was used providing new comprehensive and detailed data on cysts and encystment of a T4 genotype and Group II *Acanthamoeba* species model. This is the most common combination in human *Acanthamoeba* infections and therefore it was used also as a model in *Acanthamoeba* genome studies^21^ or in studies of the susceptibility to chemotherapeutic agents^49^. Clinical isolates used in the present study belong to species *Acanthamoeba lugdunensis* and *Acanthamoeba quina*, known as human pathogens, causative agents of *Acanthamoeba* keratitis^1,50–52^.

### Cytomorphological features of cyst wall define the Group II model species

The cytomorphological features of cysts of both strains exhibited morphology which is typical for Group II species with wrinkled ectocyst and polygonal endocyst separated by the inter-cystic space^11,20,49^. The cyst wall in both *A. lugdunensis* and *A. quina* is characteristic with the absence of superficial reticulation which can be demonstrated by silver impregnation method in some species of the Group II (e.g. *A. castellanii*)^20^. If the ectocyst possesses a distinct anastomosing and prominent network of wrinkles of 1.0–1.5 μm in thickness, the reticulation can be visualised with this method^11^. In the present isolates the surface wrinkles were detected by SEM, however, they were much narrower (0.3–0.8 μm in *A. lugdunensis* and 0.2–0.7 μm in *A. quina*). It is likely that their smaller thickness along with small thickness of the whole ectocyst prevents the intense impregnation and shows only faint traces of superficial venation that are known in all non-reticulated-cyst Group II species.

For detection of the polysaccharide-containing cyst wall by fluorescence microscopy, the staining with optical brightener is recommended in experimental conditions but also in clinical specimens^9,49^. Similarly, like in previous study^52^, the intravital staining with optical brightener Rylux, which is the Calcofluor white analogue that binds to polysaccharides, successfully demonstrated the double-layered cyst wall in both isolates. The intense fluorescence signal was detected in endocyst composed mainly of cellulose while the ectocyst showed only faint fluorescence. This result fully corresponds to the data obtained by staining of *Acanthamoeba* cysts suspensions with Calcofluor white published by Linder *et al*.^53^, Lorenzo-Morales *et al*.^47^, Kliescikova *et al*.^13^ and Moon *et al*.^54^. Contradictory results described Chávez-Manguía *et al*.^16^ who detected faint fluorescence in the endocyst and more intense fluorescence signal in ectocyst in semithin cryosections of *A. castellanii* cysts stained with Calcofluor white. Since the polysaccharide cellulose has been reported to be the major constituent of the endocyst (30%) and the ectocyst is composed mainly of proteins^37,47^, possibly this difference in fluorescence signal may be a result of fixation and extended incubation (12 h) of the cryosections with the fluorescent brightener. Further possible explanation may be the dissimilarity in thickness of cyst wall layers, where the isolate studied by Chávez-Manguía *et al*.^16^ possessed the ectocyst thicker than the endocyst.

### Cytoskeletal elements are essential in cell rearrangement during encystment

In spite of many published works on cytoskeleton in *Acanthamoeba* trophozoites, its activity during encystment process has been poorly understood with merely indirect evidences^37,38^. The presence of filamentous actin (F-actin) in *A. castellanii* cysts and its participation in the processes of encystment and excystment was studied recently by Chávez-Manguía *et al*.^19^, who observed its association with encystment vesicles in trophozoites induced to encyst and suggested its absence in the mature cysts. However, in our strains, for the first time we revealed the diffuse and patchy distribution of F-actin visualised by TRITC-phalloidin throughout entire cytoplasm of immature cysts as well as mature cysts with fully formed double-layered cyst wall. Similar results have been published for *Entamoeba invadens* cysts^55^ where higher concentrations of F-actin with patchy distribution have been noted in precyst stage, where they participate in the transportation of vacuoles and cyst wall formation. Subsequently, in mature cysts the level of polymerised actin decreased. Our results indicate that in *Acanthamoeba* cysts actin filaments persists longer than it was supposed. It can be detected also in cysts with fully formed cyst wall before its content is reduced in older cysts as it was observed by Chávez-Manguía *et al*.^19^. Previously it was showed in slime molds, that actin is involved not only in amoeboid locomotion of *Dictyostelium discoideum*^56,57^, but the activity of F-actin is considered important also in physiological maturation of spores of *Dictyostelium* with fully completed spore coat^58^.

In our study, a complex microtubular network was detected in the immature cysts of both strains for the first time. The presence of microtubules in immature cysts strongly suggests their importance in rearrangement of the cell in encystment process. Our results correspond with opinion given by Bowers and Korn^17^ who observed microtubules in encysting cells with TEM and proposed their role in cytoplasmic movements and vesicles transport leading to cyst wall synthesis. In our opinion, microtubules might function in the movement of cytoplasmic membrane-bound complexes containing the cellulose synthase and proteins involved in cellulose synthesis of the *Acanthamoeba* cyst wall that will be discussed in more detail in the next section. Such function was previously experimentally demonstrated in plants where the cortical cytoskeleton and particularly both the tubulin and actin play the most important role in the organisation of cellulose containing cell wall^59,60^. If the detected microtubules in *Acanthamoeba* immature cysts and the actin filaments in both the immature and the mature cysts may be involved not only in vesicles transport but also in the organisation of cyst wall layers remains to be elucidated in further studies.

In the present study the immunocytochemical analysis of microtubules, monomeric form of actin, and myosin in *Acanthamoeba* cysts was performed for the first time. Obtained negative results in detection of monomeric form of actin and myosin showed that these cytoskeletal elements are not present in the dormant cystic stage which agrees with the absence of cell locomotion and ceasing of organelles motility in cytoplasm.

### Cellulose fibrils are present in both layers of the cyst wall

The ultrastructure of *Acanthamoeba* cysts was studied only rarely and combined approach of transmission electron microscopy and freeze-etching methods enabled obtaining of complex and unique data that are not available for any up to the present studied *Acanthamoeba* species.

In analysed cells the observed structure of stratified ectocyst and fibrillar cellulose-containing endocyst in TEM are in accordance with literary data^13,17^. Further details of cyst wall were visualised by freeze-etching. The thickness of the ectocyst in *A. lugdunensis* (65–106 nm) corresponds to data observed in *A. palestinensis* where it was 80–150 nm^26^. In our strain of *A. quina* the thickness was even smaller (44–70 nm). However, in other species a distinctly higher thickness was recorded, in *A. astronyxis* it was 200 nm^27^, in studied strains of *A. castellanii* it reached 280–340 nm^16^ and 300–500 nm^17^, and in *A. polyphaga* it was even 650 nm^18^.

The main component of endocyst material is the cellulose^47^, giving it a fibrillar appearance in TEM. In freeze-etched specimens it has character of crossed fibrils of spiny appearance which were about 10 nm thick in studied strains. This structure corresponds well to freeze fracture data published by Spies *et al*.^29^ and Bauer^28^. Although we did not recognize the biphasic organisation of endocyst reported by Lemgruber *et al*.^18^, in both strains its structure was more loosened near the inter-cystic space than at the cytoplasmic membrane. Furthermore, the thickness of the observed endocyst in *A. lugdunensis* (210–346 nm) and in *A. quina* (100–245 nm) more or less agrees with data obtained by Chávez-Munguía *et al*.^16^ for *A. castellanii* (200–300 nm), and by Lemgruber *et al*.^18^ for *A. polyphaga* (290 nm). Interestingly, some species differ significantly in this feature. In *A. palestinensis* a very small thickness of endocyst equal to ectocyst was measured^26^ and on the other hand, in *A. astronyxis* it was significantly thicker of even 300–400 nm^27^.

The dissimilarity in thickness of cyst wall layers in *Acanthamoeba* spp. has been noted for several times with different results. As it was quoted above, in some papers the ectocyst layer was detected thicker than the endocyst, e.g. in studied strains of *A. castellanii*^16^ and *A. polyphaga*^18^. The other data on *A. astronyxis*, *A. palestinensis*^26,27^ including our results on *A. lugdunensis* and *A. quina* do not agree with these findings. However, it is supposed that thickness of cyst wall layers is dependent also on culture or environmental conditions.

Freeze-etching proved to be a method, which enables detection of scattered cellulose fibrils and small round vesicles that were not previously visualised by TEM. This way we succeeded to demonstrate for the first time the cellulose fibrils in the ectocyst that were previously not visible by any other method. Immunocytochemical methods based on selective binding of recombinant cellulose-binding protein localised the cellulose in the endocyst of *Acanthamoeba* cysts and led to conclusion that cellulose is included only in the inner layer of the cyst wall^53,61^. However, it was proved previously that cellulose in *Acanthamoeba* cyst wall is masked by non-cellulose material (mainly proteins) and without insuring the removal of masking agent by alkali treatment, the absence of cellulose cannot be demonstrated by cytochemical methods^15^. The major component of ectocyst are proteins^37,62^ that most likely may act as effective masking for lower amount of cellulose in outer cyst wall. Therefore it is likely that high amount of proteins prevented the immunocytochemical labelling of cellulose in ectocyst by recombinant cellulose-binding protein, because not any alkali treatment of cysts was performed in experiments by Linder *et al*.^53^ and Derda *et al*.^61^.

On the other hand, both the ectocyst and the endocyst of the mature *Acanthamoeba* cyst are positively stained by optical brighteners^47^ which like Calcofluor white and Rylux, are non-specific fluorochromes binding to the cellulose, chitin and possibly other polysaccharides^63,64^. Positive reaction with cellulose led to thoughts about the presence of cellulose in both layers of *Acanthamoeba* cyst wall, though without reliable evidence so far. Chávez-Manguía *et al*.^16^ suggested the presence of cellulose in the ectocyst and the endocyst on the base of semithin cryosections staining of *A. castellanii* cysts with Calcofluor white. Furthermore, Lemgruber *et al*.^18^ presupposed cellulose fibrils in the ectocyst on the base of their presence in inter-cystic space proven by quick-freeze/freeze-fracture/deep etching technique (QF-DE). In the present isolates, for the first time we found such fibrils in the ectocyst by freeze-etching method thus supporting the suggestion of Chávez-Manguía *et al*.^16^ on presence of cellulose in both layers of the *Acanthamoeba* cyst wall. Forthermore, also the method of cellulose confirmation using iodine-potassium iodide-sulphuric acid reaction was positive in both layers of the cyst wall in both strains.

It seems, that vesicles, fragments of membranes and cell debris discharged by autolysosomes are frequently deposited in the ectocyst during the encystment and may also be found in inter-cystic space near the ectocyst^16–18,26,27^. These locations were detected in our strains as well. We suppose that of the same origin could be also small electron-dense particles observed in TEM and spherical vesicles showed by freeze-etching in the endocyst material of *A. lugdunensis* strain. In both our isolates the cellulose fibrils visualised by freeze-etching were unarranged in the main part of endocyst, but in ostioles region they were distributed rather in a layer-like manner. Such stratified distribution is reported for the first time in *Acanthamoeba*. We can speculate that this slightly altered structure may serve as a reinforcement of the ostiole architecture since in this region both layers of cyst wall join and this is the place where the excystment process occurs^65^.

In *Acanthamoeba* species the layers of cyst wall are separated by electron-transparent inter-cystic space usually containing scattered fibrillar elements^17^, sometimes of spongy network appearance^26^, that we visualised well with both TEM and freeze-etching in the analysed cells of both strains. Our results support the notion, that these fibrils most likely serve as a connection between endocyst and ectocyst and in some cases they may fill almost all the inter-cystic space^18^.

Separation of endocyst from the cytoplasmic membrane by an electron-transparent space in mature cysts observed in both our strains is typical feature of mature *Acanthamoeba* cysts. Our findings of small electron-dense particles dispersed in this space and a pale amorphous layer covering the cytoplasmic membrane support the opinion given by Bowers and Korn^17^ who assumed that this zone represents an additional amorphous layer. Probably the thickness of this zone depends on the age of cyst and may extend due to advancing dehydration during encystment.

### Clusters of intramembranous particles (IMPs) in cytoplasmic membrane support the proposed hypothesis of cellulose microfibril terminal complexes (TCs) involved in cellulose synthesis during encystment

In the present study, the freeze-etching method allowed obtaining of some new information on the encystment process in *Acanthamoeba*. In some cytoplasm regions the production and release of vesicles from the cortical cytoplasm was noted. The function of these vesicles is unclear. Previously it was supposed, that cytoplasmic vesicles with electron-dense filamentous content observed under TEM during the encystment of *A. castellanii* in the cortical cytoplasm may contain a cellulose-like material and participate in the cyst wall formation^16,18^. However, the autoradiographic study of immature *A. castellanii* cysts revealed earlier, that the participation of Golgi complex in the cyst wall synthesis is limited to proteins and oligosaccharides but in Golgi vesicles the cellulose is not present^66^. The mechanisms associated with cellulose biosynthesis in *Acanthamoeba* remain unclear and it is assumed that cellulose synthesis may occur similarly like in algae and higher plants, or in spores of the slime mold *Dictyostelium discoideum*. In *Dictyostelium*, prespore vesicles (PSVs) participate in the spore coat formation in release mainly of proteins and other polysaccharides via exocytosis but the cellulose synthesis occurs directly at cytoplasmic membrane by cellulose synthase enzyme complex utilizing cytoplasmic UDP-glucose^67^. The same process of cellulose microfibrils formation is suggested also in *Acanthamoeba*^68^. Therefore it is likely, that cortically located vesicles observed in *Acanthamoeba* may serve in the similar way like PSVs in *Dictyostelium*, i.e. in excretion of other components of cyst wall.

In *Acanthamoeba* we observed considerable alterations of the density of intramembranous particles (IMPs) in the protoplasmic fracture face (PF) and exoplasmic fracture face (EF), and alterations of IMPs size in the PF of the cytoplasmic membrane during the encystment. In immature cysts producing the endocyst, a high density of IMPs in PF (1059 ± 77 particles/μm^2^ in *A. lugdunensis* and 2056 ± 659 particles/μm^2^ in *A. quina*, mean ± SE) with tendency to cluster into diads, triads, tetrads or even more was detected in cytoplasmic membrane. In mature cysts possessing the completed cyst wall, their size and density considerably decreased to about 40% *A. lugdunensis* and to about 3–10% in *A. quina*. Similarly, the density of IMPs in EF of the cytoplasmic membrane in immature cysts was conspicuously high (7793 ± 890 particles/μm^2^ in *A. lugdunensis* and 3074 ± 810 particles/μm^2^ in *A. quina*, mean ± SE) and in mature cysts it decreased approximately to 25% in *A. lugdunensis* and to 28% in *A. quina*. Our findings are in accordance with earlier results by Spies *et al*.^29^ who described tendency of IMPs clustering in cytoplasmic membrane of encysting *Acanthamoeba* cells and a reduction of their density, size, and differentiation in mature cysts. However, they did not provide any further details on values, and moreover, data on size or density of IMPs are lacking in other publications on cysts of acanthamoebae. Similar changes in the cytoplasmic membrane were described in the slime mold *Physarum polycephalum* where the IMPs density decreases during the sporulation^69^. These findings support the interpretation of metabolic activity decline in dormant stages of protists.

The only published values on IMPs density in the cytoplasmic membrane of *Acanthamoeba* concerns trophozoites where Bowers^70^ noted remarkably low density (in PF 200–400 particles/μm^2^, in EF 100–200 particles/μm^2^) in comparison with the cysts from our experiments. From all these results including our findings can be deduced, that at the beginning of *Acanthamoeba* encystment, the density of IMPs in the cytoplasmic membrane rises up to the stage of endocyst synthesis (culmination of cellulose synthesis) in the immature cyst. Subsequent decrease of IMPs density follows the cyst wall completion in mature cysts. It is obvious that the higher density of IMPs positively correlates with the higher metabolic activity of the membrane^70^ and it gives us an interpretation of the changes in IMPs density.

**Figure 9:**
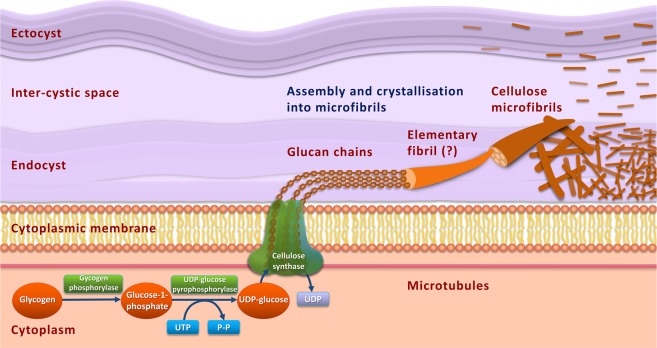
Hypothetical model for the biosynthesis of cellulose in *Acanthamoeba* cyst. Glycogen in the cytoplasm is degraded by catalytic action of glycogen phosphorylase, releasing glucose-1-phosphate. Subsequently, UDP (uridine diphosphate) glucose is synthesised from glucose-1-phosphate and UTP (uridine triphosphate) catalysed by UDP-glucose pyrophosphorylase. Through the action of cellulose synthase the glucose residues are polymerized into individual glucan chains that are translocated across the cytoplasmic membrane. The interaction of the cellulose synthase with microtubules is strongly supposed. Glucan chains are likely assembled into elementary fibrils that are associated into microfibrils. This process is called crystallisation. For simplicity, the number of glucan chains is schematic.

The cellulose is usually synthesised by membrane-bound complexes that can be visualised by freeze techniques as groups of intramembranous particles^71^. These cellulose microfibril terminal complexes (TCs) contain cellulose synthase and proteins required for the deposition and crystallisation of cellulose outside the cytoplasmic membrane in bacteria, plants^72^ and in spores of *Dictyostelium discoideum*^73^. On the base of our findings, we propose a hypothesis, that similarly in *Acanthamoeba* during the encystment, the IMPs clusters found in cytoplasmic membrane represent cellulose microfibril terminal complexes (TCs) involved in cellulose synthesis. Their subsequent reduction in mature cysts is a result of the cyst wall completion with cellulose synthesis terminated. The hypothetical model of cellulose synthesis in *Acanthamoeba* is shown in Fig. 9. As the precursor of cyst wall cellulose serves cellular glycogen^66^ which is degraded by catalytic action of glycogen phosphorylase. Glucose residues are polymerized by the catalytic action of cellulose synthase into individual glucan chains that are translocated across the cytoplasmic membrane and assembled into microfibrils that are deposited in both layers of the cyst wall. We think that similarly as it is known in plants^59,72^, the deposition of microfibrils may be guided by microtubules which we detected in the immature *Acanthamoeba* cysts. These microtubules may be adjacent to, or directly connected with, the cellulose synthase complex. In the immature cysts we detected a complex network of unarranged microtubules which strongly correlates with unarranged crossed cellulose fibrils in endocyst. This way the cytoskeleton regulates the formation of cellulose in the cyst wall.

During the encystment the *Acanthamoeba* cell undergoes considerable dehydration which can result in around 80% decrease of cell volume and 65% decrease of surface area. An increased rate of water discharge occurs mainly during the retraction of the cell from finished ectocyst and is followed by formation of the endocyst^17^. We documented this stage of immature cyst double-labelled with anti-α-tubulin and phalloidin-TRITC showing contractile vacuole that is the most responsible element in this process of retraction^17^. Using the freeze-etching, we observed in the endocyst synthesising immature cysts the cytoplasmic membrane closely adherent to endocyst whereas in mature cysts of *A. lugdunensis* their separation was noted. Simultaneously with significant reduction of cytoplasmic membrane area, these processes resulted in its deformation to numerous wrinkles and invaginations that we detected in mature cysts. Performed TEM analysis well corresponded with tridimensional examination by freeze-etching which excellently illustrated the real appearance of cytoplasmic membrane. Similar invaginations detected Lemgruber *et al*.^18^ using QF-DE technique who assumed that the irregularity of cytoplasmic membrane of encysted cell is caused by the presence of secreted vesicles associated with cell wall synthesis. However, we did not detect such process in our cells and we suggest the dehydration as the most responsible factor for the wrinkles formation.

Progressive dehydration, which we observed in the course of encystment process, manifested in higher degree of cytoplasm condensation with a maximum in mature cysts. These changes were connected with alterations in structure of nucleus. With the use of freeze-etching technique, we detected advanced stage of dehydration of nucleoplasm, decrease of nuclear membrane differentiation from cytoplasm, and distinct reduction (of about 11%) of nuclear pore complexes size. Although the condensation of cytoplasm and some morphological changes of nuclei in encysting cells were previously described in TEM^17,26^ our study brings the first report on changes in the nuclear membrane in *Acanthamoeba* as visualised by freeze-etching. Interestingly, in *Entamoeba histolytica* the size of nuclear pore complexes does not alter during the encystment and remains in values of about 79 nm in diameter^74^ that is similar to our measurements in immature cysts.

In the analysed cells, numerous spherical lipid droplets were noted in the cortical cytoplasm of the cell closely under the whole cytoplasmic membrane in interference contrast, TEM and freeze-etching. It is known that the amount of lipid droplets increases during the encystment and takes cortical position in mature *Acanthamoeba* cysts^17,75^. During the encystment also morphological alterations of mitochondria including changes in shape and tubular cristae occur. In mitochondria, we found internal lamellar structures and strongly electron-dense crescent-like bodies located on the periphery that correspond to similar structures described by Bowers and Korn^17^ and Lasman^26,27^ who supposed their origin as a result of respiration decrease during the encystment. Similar electron-dense inclusions were found in hydrogenosomes which evolved from mitochondria in anaerobic free-living and parasitic protists, and anaerobic fungi. High content of calcium and phosphate was detected by energy dispersive X-ray microanalysis in these inclusions in hydrogenosomes of the ciliate *Metopus contortus*^76^ and anaerobic fungus *Neocallimastix frontalis*^77^. Therefore we may suppose a similar structure in *Acanthamoeba* mitochondrial inclusions, i.e. probably hydroxyapatite deposits, as well.

### Disruption of cellulose synthesis pathway seems to be promising in the treatment of *Acanthamoeba* infections

Our results proved the cellulose fibrils as a structural component of both layers of the cyst wall. This stresses once again the importance of the cellulose as a target in strategies against acanthamoebae. At present, cellulose biosynthesis pathway is considered to be a potential target in treatment of *Acanthamoeba* infections and disruption of this pathway would prevent the first steps of encystment, i.e. synthesis of the cyst wall and this way opens the admission of biocides to the cell^43,45^. Lorenzo-Morales *et al*.^47^ showed that the silencing of the glycogen phosphorylase expression substantially disrupted the cyst wall assembly and vast majority of treated amoebae was then eliminated by 0.5% sodium dodecyl sulphate (SDS) which was completely ineffective to eliminate untreated mature cysts. Further experiments demonstrated that silencing of the cellulose synthase with siRNA probe blocks the encystment of more than 50% of acanthamoebae in culture^48^. These results may represent the possible way of how to reduce considerably the resistance of cysts which would be then more susceptible to therapeutic agents. It seems that the combined therapy including the disruption of cellulose biosynthesis pathway alongside the application of chemotherapeutic agents may be a promising approach in treatment of *Acanthamoeba* infections.

## Conclusions

Pathogenic acanthamoebae of the T4 genotype and Group II species are the most frequent causative agents of *Acanthamoeba* infections. The cyst stage defines the resistance of acanthamoebae to disinfectants and chemotherapeutic agents and leads to serious difficulties in their elimination in environment and invaded tissues as well. The development of new strategies against acanthamoebae focuses on the cyst wall and especially on the cellulose synthesis and degradation. However, understanding of involved biological and biochemical processes correlates highly with understanding of the cytomorphology and ultrastructure and from this point, the cysts and the encystment process in *Acanthamoeba* are still insufficiently investigated. The elucidation of processes involved in cyst wall formation may contribute to develop new treatment approaches of *Acanthamoeba* infections.

## Materials and Methods

### Amoeba strains

Two clinical isolates of the genus *Acanthamoeba* belonging to T4 genotype were used in the study. The strain of *Acanthamoeba lugdunensis* Pussard & Pons, 1977 was isolated from a corneal scrape from a severe case of keratitis with coinfection with *Pseudomonas aeruginosa* in the left eye^50^. The strain of *Acanthamoeba quina* Pussard & Pons, 1977 was isolated from a corneal scrape from a patient with a keratitis and scleritis in the right eye^52^.

### Growth conditions

Acanthamoebae were cultured axenically in peptone-yeast extract-glucose medium (PYG) (proteose peptone from animal tissue and yeast extract were produced by Sigma-Aldrich, Slovak Republic). Axenic cultures were obtained as described previously^52^. Briefly, from the 2-day monoxenic cultures on agar plates (1.5% non-nutrient agar with a layer of *E. coli*), the trophozoites were axenised by inoculation into the Bacto-Casitone/Serum medium (BCS) with penicillin and ampicillin in order to eliminate bacteria. After 72 h, the active trophozoites were transferred into PYG medium with penicillin and ampicillin. After 5 passages, the trophozoites were transferred into a PYG medium without antibiotics and subsequently cultivated in this medium with subcultivation once a week. The cultures were incubated at 37 °C.

### Encystment

For encystment of the amoebae, active trophozoites in PYG medium were inoculated on the surface of agar plates (without bacteria). After 7 days, the cysts were rinsed out from the agar surface with a sterile AS solution, which is a modified Neff’s amoeba saline^11^. For the subsequent analyses, the cells were concentrated by centrifugation at 800 × g for 20 min and subsequently resuspended in PYG medium.

### Light microscopy analysis

The morphology of live cysts was observed and documented in hanging drop preparations. The measurements were performed on vital cysts. In total, 25 cysts were measured for each strain on the object slide to obtain morphometric data.

### Iodine staining of cyst wall and iodine-potassium iodide-sulphuric acid cellulose test

A drop of iodine-potassium iodide solution was mixed with suspension of unfixed cysts on the cover slips and immediately observed and documented in hanging drop preparations. For the cellulose test the same procedure was done with subsequent addition of a drop of 70% sulphuric acid^78^. After preparing the hanging drops, the suspensions were immediately observed and documented.

### Silver impregnation

Cysts suspensions were mixed with glycerol–albumin on glass slides and fixed for 2 h by Clarke’s fixation mixture^11^. After a short rinse in distilled water, the samples were impregnated in 0.5% silver proteinate (protargol) for 2 h at 60 °C, then briefly (several seconds) immersed in developer (1% hydroquinone in 5% sodium sulphite), subsequently rinsed in distilled water, dehydrated and mounted on slides in Dammar resin or Eukitt (O. Kindler, Germany).

Digital images were captured at laboratory temperature under bright-field illumination and differential interference contrast (DIC) on Zeiss Axioskop 2 microscope with an oil-immersion Plan-Neofluar 100×/1.30 NA objective lens (Carl Zeiss, Germany), equipped with DP72 12.8 Mp colour camera (Olympus, Japan) using DP Capture software (Olympus, Japan). Raw TIFF images were assembled using Photoshop CS5 (Adobe, USA). Identical adjustments in brightness and contrast were applied to all images in a given experiment.

### Fluorescent staining of cyst wall cellulose

For visualisation of cyst wall, the intravital staining with a fluorescent brightener, Rylux (Ostacolor, Czech Republic) which is a diaminostilbenedisulphonic acid derivate, was used^52^. Briefly, the stock solution was prepared by dissolving Rylux in modified Neff’s amoeba saline^11^ to a concentration of 10^−2^%. A drop of Rylux stock solution was mixed with suspension of unfixed cysts on the cover slips. After preparing the hanging drops, the suspensions were immediately observed with a fluorescence microscope Leica DM 2500 equipped with HC PL Fluotar 100×/1.32 NA oil-immersion objective (Leica, Germany). Digital images were captured with EOS600D 18 Mp camera (Canon, Japan) using the EOS Utility Capture software (Canon, Japan) at laboratory temperature. Raw TIFF images were assembled using Photoshop CS5 (Adobe, USA). Identical adjustments in brightness and contrast were applied to all images in a given experiment.

### Fluorescent staining of cell proteins for confocal laser scanning microscopy

Modified protocols for localisation and visualisation of cytoskeletal proteins and motor protein myosin followed Valigurová^79^. Cell suspensions were fixed on glass slides for 15 min in freshly prepared 4% paraformaldehyde in 0.2 M phosphate buffered saline (PBS) at room temperature, subsequently washed, and permeabilised for 10 min in 0.3% Triton X-100 (Sigma-Aldrich, Czech Republic).

For direct fluorescent staining, samples were washed three times in 0.1 M PBS for 10 min, incubated with phalloidin–tetramethylrhodamine B isothiocyanate (phalloidin-TRITC; Sigma-Aldrich, Czech Republic) overnight at room temperature and then washed again in PBS. For indirect immunofluorescent analysis, samples were incubated overnight at 4 °C in mouse monoclonal anti-α-tubulin antibody (Clone B-5-1-2, Sigma-Aldrich, Czech Republic; dilution 1:1000) or in mouse monoclonal IgG anti-actin antibody (1:10) that was raised against *Dictyostelium* actin (provided by Prof. Dominique Soldati-Favre) diluted in PBS with 0.1% bovine serum albumin (BSA), washed three times in PBS for 10 min and incubated with FITC-conjugated anti-mouse polyvalent immunoglobulins (1:125) in PBS with 1% BSA at 37 °C for 4 h. Samples incubated with rabbit anti-myosin antibody (smooth and skeletal, the whole antiserum, Sigma-Aldrich, Czech Republic; dilution 1:5) overnight at 4 °C were after washing (three times in PBS for 10 min) incubated with TRITC-conjugated anti-rabbit IgG (whole molecule) (1:200) in PBS with 1% BSA at 37 °C for 4 h. All samples were counterstained with Hoechst 33258 (Molecular Probes, USA) for 30 min at room temperature and subsequently washed in PBS. Controls were labelled with FITC-conjugated secondary antibody or TRITC-conjugated secondary antibody alone without the primary antibody.

Preparations were mounted in anti-fade mounting medium based on 2.5% DABCO (Sigma-Aldrich) mixed with glycerol and 0.1 M PBS, analysed and documented at laboratory temperature using an Olympus IX81 FVBF-2 microscope with an oil-immersion UPlanApo 100×/1.35 NA Oil Iris objective lens (Olympus, Japan), equipped with a laser-scanning FluoView 500 confocal unit and DP70 12.5 Mp camera (Olympus, Japan) using FluoView 4.3 software (Olympus, Japan). Fluorescence was visualised using the TRITC (phalloidin, anti-myosin), FITC (anti-actin, anti-α-tubulin) and/or UV (Hoechst) filter sets. Raw TIFF images were assembled using Photoshop CS5 (Adobe, USA). Identical adjustments in brightness and contrast were applied to all images in a given experiment. All experiments were performed repeatedly.

### Scanning electron microscopy

The suspensions of cysts were sedimented by centrifugation and fixed in freshly prepared 2.5% glutaraldehyde in 0.2 M cacodylate buffer (pH 7.2). Specimens were then washed 3 × 15 min in the cacodylate buffer, post-fixed in 2% osmium tetroxide in cacodylate buffer for 2 h at room temperature, and finally washed 3 × 15 min in the same buffer. After dehydration in ascending acetone series, specimens were critical point-dried using CO_2_, coated with gold. The material was examined and images were acquired at laboratory temperature with JSM-7401F field emission scanning microscope (Jeol, Japan) operating at 4 kV^80^. Raw TIFF images were assembled using Photoshop CS5 (Adobe, USA). Identical adjustments in brightness and contrast were applied to all images in a given experiment.

### Transmission electron microscopy

For preparation of ultrathin sections two alternative procedures were performed. In the first approach, a small block (1 mm^3^) of agar with adhered cysts was cut from the plate and fixed with 2% glutaraldehyde in 0.075 M cacodylate buffer (pH 7.2) for 2.5 h, at laboratory temperature. Fixed cysts were washed in 0.075 M cacodylate buffer and postfixed in 1% osmium tetroxide in the same buffer overnight, at 5–6 °C. Subsequently, the material was washed, dehydrated in ascending ethanol series and embedded in Spurr resin^81^.

Alternatively, the suspension of cysts was sedimented by centrifugation (2500 rpm), and inoculated into a drop of freshly prepared 2% agar (42 °C). A block of agar (1 mm^3^) with embedded cysts was cut and fixed with 2.5% glutaraldehyde in 0.2 M cacodylate buffer (pH 7.2) overnight, at 4 °C. Fixed cysts were washed in 0.2 M cacodylate buffer and postfixed in 1% osmium tetroxide in the same buffer for 3 h. Subsequently, the material was washed, dehydrated in ascending ethanol series and embedded in Epon (Polybed 812)^80^.

Ultrathin sections were stained with uranyl acetate, followed by lead citrate. Observations and images acquisition were made at laboratory temperature using a Jeol JEM 2000FX TEM electron microscope operating at 80 kV. Images were acquired with photo-equipment of the microscope on negative sheet film and after developing they were scanned as raw TIFF images which were assembled using Photoshop CS5 (Adobe, USA). Identical adjustments in brightness and contrast were applied to all images in a given experiment.

### Freeze-etching

Cyst suspensions were fixed overnight at 4 °C in freshly prepared 2.5% glutaraldehyde in 0.2 M cacodylate buffer (pH 7.2) followed by cryoprotection with 20% glycerol treatment overnight at 4 °C in the fridge. Subsequently the dense suspension was put on the gold carrier and frozen in melting liquid nitrogen (−210 °C). The frozen samples were transported into the working chamber of the freeze-etching system device BAF 060 (BalTec, Switzerland). The freeze-fracture process was done by microtome knife cooled on very low temperature and rotated at 60 rpm, the sample holder temperature was set on −147 °C. During the process of etching the temperature of sample holder was set on −100 °C for 20 min with the pressure inside the working chamber of 10^−5^ Pa. Replicas were prepared by vacuum deposition of platinum (angle of evaporation 45°, thickness of layer 2.4 nm) and carbon (angle of evaporation 90°, thickness of layer 22.2 nm) onto the frozen and fractured surface. The replicas were cleaned with sodium hypochlorite, chromo-sulphuric acid and sulphuric acid to remove all the biological material, and washed in distilled water. The pieces of replica were mounted on copper grids and examined under Morgagni 268D, FEI transmission electron microscope (Philips, Netherlands). Images were acquired at laboratory temperature with photo-equipment of the microscope as raw TIFF images, which were assembled using Photoshop CS5 (Adobe, USA). Identical adjustments in brightness and contrast were applied to all images in a given experiment. Statistical evaluation of intramembranous particles (IMPs) per a unit area (1 μm^2^) was done in ImageJ software, and histograms illustrating the IMPs size distribution were prepared in Microsoft Office Excel 2007 (Microsoft Corporation, USA).

## Data Availability

The data contributing to this study are included in the manuscript. Supporting data are available from the corresponding author on reasonable request.
